# Impacts of the COVID-19 Lockdown on Healthcare Inaccessibility and Unaffordability in Uganda

**DOI:** 10.4269/ajtmh.23-0144

**Published:** 2023-08-14

**Authors:** Bijetri Bose, Shamma A. Alam, Claus C. Pörtner

**Affiliations:** ^1^ Fielding School of Public Health, University of California, Los Angeles, California;; ^2^ Department of Global Health and Population, Harvard T. H. Chan School of Public Health, Boston, Massachusetts;; ^3^ Department of International Studies, Dickinson College, Carlisle, Pennsylvania;; ^4^ Department of Economics, Seattle University, Seattle, Washington;; ^5^ Center for Studies in Demography and Ecology, University of Washington, Seattle, Washington

## Abstract

Several studies have reported adverse consequences of the COVID-19 lockdowns on the utilization of healthcare services across Africa. However, little is known about the channels through which lockdowns impacted healthcare utilization. This study focuses on unaffordability as a reason for not utilizing healthcare services. We estimate the causal impacts of the COVID-19 lockdown on healthcare inaccessibility and affordability in Uganda relative to the nonlockdown periods of the pandemic. We use nationally representative longitudinal household data and a household fixed-effects model to identify the impact of the lockdown on whether households could not access medical treatment and whether the reason for not getting care was the lack of money. We find that the lockdown in Uganda was associated with an 8.4% higher likelihood of respondents being unable to access healthcare when treatment was needed relative to the nonlockdown periods. This implies a 122% increase in the share of respondents unable to access healthcare. As lockdown restrictions eased, the likelihood of being unable to access medical treatment decreased. The main reason for the increase in inaccessibility was the lack of money, with a 71% increase in the likelihood of respondents being unable to afford treatment. We find little evidence that the effects of the lockdown differed by wealth status or area of residence. Our results indicate the need for policymakers to consider immediate social support for households as a strategy for balancing the disruptions caused by lockdowns.

## INTRODUCTION

Many countries implemented large-scale lockdowns of their populations from 2020 to 2021 to control the pandemic caused by the coronavirus SARS-CoV-2. This nonpharmacological intervention to control the COVID-19 disease involved minimizing social contact by closing schools, workplaces, restaurants, religious buildings, and recreational areas; restricting mass gatherings and inter- and intrastate travel; suspending international flights; canceling public events and public transport; and requiring people to stay home.^1^^,^^2^ Although such measures effectively mitigated the pandemic,^3^ the lockdowns disrupted the lives of millions of people globally and raised serious concerns about access to essential services, such as healthcare. Although declining access to healthcare is of concern everywhere, lack of healthcare access likely presents a particular problem in Africa because of its already weak healthcare systems and a population vulnerable to infectious diseases.^4^

Several studies have reported on the adverse consequences of the lockdown on the utilization of non-COVID-related healthcare services globally.^5^^–^^11^ These studies differ from the broader literature on the impact of the COVID pandemic on healthcare access by focusing on the effects of lockdowns on healthcare utilization. However, there has been relatively little investigation into the channels through which lockdowns impacted healthcare utilization. Most studies using advanced statistical methods, such as interrupted time series or difference-in-difference, to examine the link between lockdowns and access to care in Africa have discussed the potential reasons for the reduced access without empirically testing them. The reasons suggested include difficulties in getting to health facilities as a result of lack of transportation, police violence, and insufficient money^5^^,^^6^^,^^12^^–^^14^; uncertainty about service availability at facilities because of stock outages and reduced operating hours^5^^,^^6^^,^^13^; lack of clarity on what constituted essential travel and the need of proof^6^; and inability to pay for healthcare services because of a drop in household income.^12^

From a policy perspective, it is vital to quantify the role of these reasons in the disruption of access to healthcare due to the lockdown. Although all reasons likely had some effect, assessing the impact of the above-stated reasons will allow policymakers to understand the pros and cons of lockdowns as a public health tool to control pandemics and design alleviating policies in case lockdowns are used. In this study, we focus on unaffordability as a reason for not utilizing healthcare services. Studies have shown that lockdowns implied lower household income and a decline in remittances,^15^^–^^19^ which, in turn, are likely to have reduced care seeking in several African countries because of the lack of money for healthcare services, especially in the private sector, and for transportation.

The problem of unaffordability is particularly relevant in Africa, where direct payments by patients at the point of care is the predominant method of meeting health expenditures and a source of financial risk to households.^20^^–^^23^ In Uganda, out-of-pocket payments for healthcare services are dominant, accounting for 40% of the country’s total health expenditure.^24^ Almost 3% of Ugandans were pushed below the national poverty line as a result of health-related expenses in 2016–2017.^24^ Even with the abolition of all fees at primary care government health facilities in 2001, catastrophically high health expenditure continues to plague patients as a result of inefficiencies in the system, such as informal payments to healthcare workers and the unavailability of drugs at government facilities that drives patients to the more expensive private facilities.^25^

We estimate the causal impacts of Uganda’s first COVID-19 lockdown on healthcare utilization and affordability relative to nonlockdown periods of the pandemic. By comparing lockdown periods to nonlockdown periods using countrywide panel data with household fixed-effects models, we can control for the impact of the pandemic and establish a causal relationship between the lockdown and the channels through which it impacts healthcare utilization. Lockdowns have been used previously in African countries and continue to be used as a public health tool, such as during the 2022 Ebola outbreak in Uganda, where a 21-day lockdown was implemented.^26^ The findings of this paper contain essential lessons for those cases.

Although many African countries implemented lockdowns, we use Uganda as a case study because it had one of the strictest and longest lockdowns.^27^^–^^29^ This allows us to focus on the consequences of the lockdown without worrying about to what extent the rules were implemented. Furthermore, Uganda shares characteristics with many other countries in the continent, particularly in the sub-Saharan African region, in terms of several economic indicators, making the findings of this paper generalizable to other countries.

In addition to examining whether unaffordability of care during the lockdown was a primary channel through which the lockdown disrupted healthcare utilization, our study makes two significant contributions to the literature. First, we use data from a nationally representative household survey in Uganda. This is unlike most studies, which rely on data with a narrow geographical focus, covering a few cities to one province in a country at most.^5^^,^^6^^,^^9^^,^^10^ Second, the household survey allows us to consider access to medical treatment across all healthcare providers and facilities. All existing rigorous studies based on African data, including one study from Uganda,^11^ rely on data from public healthcare facilities.^5^^,^^6^^,^^9^^,^^10^ This sampling is problematic because a large share of healthcare access in Africa involves the private sector, including private practitioners, informal providers, and pharmacies.^30^^–^^32^

## MATERIALS AND METHODS

### Study setting.

On March 18, 2020, the Ugandan government started imposing restrictions, including travel restrictions and cancellation of public gatherings such as religious services, weddings, and music events.^33^ A total lockdown was imposed on March 30 with a nationwide curfew from 7:00 pm to 6:30 am, a ban on public transportation, strict regulations on the movement of vehicles, and closure of all nonessential businesses, which lasted until the end of May.^34^^,^^35^ Lockdowns were eased at the beginning of June 2020 with the resumption of public transportation and the opening of businesses.^36^^–^^39^ Most small and medium businesses were reopened by July–August 2020.^34^ International travel remained restricted until the end of September, when land borders reopened and international flights resumed.^36^

Healthcare delivery in Uganda is provided by a mix of public and private providers that is categorized into six levels, ranging from national referral hospitals providing specialist care to health promotion activities at the community level.^40^ The government provides formal care at all levels, with primary care being free, whereas the private sector is more diverse, with a range of formal and informal providers.^30^^,^^41^ The utilization of healthcare services has improved over time in the country. For example, approximately three-fourths of live births from 2011 to 2016 occurred in a health facility, and over 80% of children with a fever or acute respiratory infection received advice or treatment at a facility or provider.^42^ However, the healthcare system in Uganda has been criticized for being inequitable, with “getting money for treatment” and “distance to facility” being frequently documented barriers^43^; 45 and 37% of women, respectively, reported these problems with healthcare access in the 2016 Demographic and Health Survey.^42^

### Data.

We use nationally representative longitudinal household data from the Uganda High-Frequency Phone Survey on COVID-19 (UHFS) conducted by the Uganda Bureau of Statistics in collaboration with the World Bank. The objective of the survey was to provide insights into the economic and social impacts of the COVID-19 pandemic.^33^ It has been conducted regularly from June 2020 to August 2022. Because we are interested in the periods during and immediately after the first lockdown in Uganda, we use the first six rounds of the UHFS conducted from June 2020 to April 2021. Four of the six rounds were in 2020 (June, August, September, and October), and two were in 2021 (February and March).

The UHFS sample is a subset of the 3,098 households interviewed in the eighth wave of the Uganda National Panel Survey in 2019/2020 (UNPS 2019/20). In the UNPS 2019/20, respondents were requested to provide a phone number. Initially, the goal was to ensure households could be reached in case they moved, but the phone numbers became the basis for surveying households during the pandemic. Because phone survey respondents differ from the general adult population, we use the sampling weights from the phone survey to make our analysis nationally representative.^44^ The phone survey weights were based on the original UNPS but were adjusted to reflect the selection and interview process.^33^ The weighted summary statistics of the households sampled in the UNPS and UHFS match closely, indicating that relying on phone numbers is unlikely to have biased the sample used in this study.^33^

Of the 2,386 households that provided a phone number, 2,227 were successfully interviewed for round 1 of the UHFS. Over the six rounds, the attrition rate was 5.7%, with 2,100 households from the baseline interviewed in round 6. However, replacement households were added to the sample after the first round, bringing the total sample size to 2,186. We excluded households with missing data on the outcome and control variables and ended up with a final sample of 2,041 households.

### Outcomes.

In all survey rounds, the UHFS asked the respondents whether any household member could access medical treatment, conditional on the respondent stating there was a need for treatment. On the basis of this question, we created a binary variable for inaccessibility of medical treatment, where 1 indicates that the respondent could not receive medical treatment and 0 otherwise.

For those unable to access medical treatment, a follow-up question asked about the main reasons that medical treatment was not accessible. On the basis of the response, we created a second binary variable indicating unaffordability as the main reason for not accessing healthcare, which takes the value 1 when the respondent selected “lack of money” and 0 otherwise. Note that lack of money could refer to insufficient money to pay for healthcare services or travel to facilities.

The other response options correspond to two broad reasons for not getting treated: facility-related issues and travel-related issues. The facility-related issues included the following options: “no medical personnel available,” “turned away because the facility was full,” “fear of getting infected at the health facility,” “hospital/clinic not having enough supplies or tests,” and “facility was closed.” The travel-related issues included “did not get clearance from the authorities to travel to a facility,” “facility too far,” and “lack of transportation.”

The time frame for the question on medical treatment varied across the surveys. The first round of the survey asked whether any household member needed medical treatment from the start of the lockdown (March 20, 2020, to June 2020; a period of 10 weeks between the beginning of the lockdown and the start of interviews). The later surveys collected information on medical treatment since the previous survey, with the surveys typically conducted at 4-week intervals.

**Figure 1.**
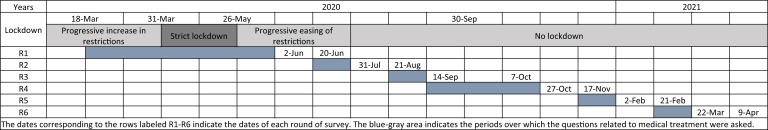
Timing of the COVID lockdown and surveys. R = round.

Uganda was under varying stages of lockdown from March 18 to July/August 2020. Hence, any reports on medical treatment in the first survey round—which contained information between March 20, 2020, and June 2020—correspond to the need for medical treatment during the lockdown. The data from the first round represent an initial period of strict restrictions followed by their gradual easing. Because the second survey round asked about healthcare needs from June 20, 2020, to July/August 2020, data from this round represent an increasingly relaxed period of the lockdown. Using the first two rounds individually ensures a nuanced understanding of the differing effects of the strict and relaxed phases of the lockdown. Rounds 3–6 correspond to the nonlockdown periods. Figure 1 details the timing of lockdowns and each survey round.

### Statistical analysis.

To establish the causal effects of the COVID-19 lockdowns, we use household fixed-effects models on the UHFS dataset. We rely on the changes over time in government-imposed lockdowns to identify the effect. We quantify the impact of the lockdown on the inaccessibility and unaffordability of healthcare by estimating the following equation:

$$Y_{i,t}=\beta_{0}+\beta_{1}L+\beta_{2}{\text{Cases}}_{i,t}+\beta_{3}X_{1\;i,t-1}+\delta_{i}+\varepsilon_{i,t}\text{,}$$ (1)

where *Y_i,t_* denotes the two outcomes of interest for household *i* in survey round *t*. *L* is a binary variable indicating the lockdown-related period, with 1 representing a lockdown-related period and 0 otherwise. It represents the first and second survey rounds. Our estimations compare the periods during the lockdown to those with no lockdowns in rounds 3–6, with observations from these rounds being pooled. We also estimate Equation (1) by separating *L* into two periods corresponding to the initial and stricter period of the lockdown (*L_1_*), captured through the first round of the survey, and the later, more relaxed period of the lockdown (*L_2_*), captured through the second round. This allows us to assess the lockdown’s impact at different intensity stages on healthcare utilization.

In addition to government-imposed lockdowns, individuals may modify their health-seeking behaviors if they perceive a high risk of contracting COVID-19. To capture the severity of the COVID situation, the Cases variable measures the number of new COVID-19 cases per 100,000 persons in the 30 days before the household’s survey date. The number of COVID cases comes from Our World in Data.^45^ Lockdowns may also influence the household structure, so *X_1_* represents the number of household members in the prior round. We use the lagged values from the previous survey round to reduce endogeneity concerns.

The household fixed effects, *δ_i_*, control for unobserved household-level time-invariant factors that may bias the results. This approach allows us to control for time-invariant characteristics associated with the individual/household, such as gender, race and religion, constant preferences, household characteristics, and area characteristics, and other time-invariant factors.

## RESULTS

### Summary statistics.

The households in the sample had an average of five members, with a mean of 2.4 males and 2.6 females. The majority of the households were male headed (69%) and in rural areas (68%). We first present evidence on the trends in access to medical treatment from the first six rounds of the UHFS. Table 1 shows the percentage of respondents who reported being unable to access medical treatment over the six rounds. Of those needing medical treatment, 17% (236/1,354) reported being unable to access care in round 1. The share of such respondents declined to 10% (133/1,284) in round 2, which came after the progressive easing of the restrictions. The decline continued over the next rounds before slightly rising to 7% (69/1,027) in round 6.

**Table 1:** Trends in inability to access medical treatment and its associated reasoning

Access indicators		R1	R2	R3	R4	R5	R6
Needed medical treatment	*n*	1,354	1,284	1,162	1,246	1,379	1,027
Unable to access treatment	*n*	236	133	109	83	72	69
	%	17	10	9	7	5	7
Reasons for not accessing medical treatment							
Unaffordability	*n*	157	117	102	75	65	57
	%	66.5	88.0	93.6	90.4	90.3	82.6
Facility-related issues	*n*	13	2	2	3	3	1
	%	5.5	1.5	1.8	3.6	4.2	1.4
Travel issues	*n*	55	5	0	0	1	0
	%	23.3	3.8	0.0	0.0	1.4	0.0
Total number of households	*N*	2,223	2,146	2,098	2,065	2,042	2,041

R = round. R1–R6 represent the six survey rounds used in the analysis. *n* indicates the frequency of respondents reporting being unable to access care or reporting a reason for not accessing medical treatment in each round. *N* is the total number of households.

Lack of money was the primary reason for those who could not access medical treatment. As shown in Table 1, of those unable to get care in round 1, 67% (157/236) attributed it to lack of money, whereas 23% (55/236) reported travel-related issues. As travel restrictions were lifted in later rounds, reporting of travel-related issues decreased to 0% in rounds 3, 4, and 6.

### Effect on accessibility and affordability.

Table 2 reports the impact of the lockdowns on the inaccessibility and unaffordability of medical treatment. The results in column 1 indicate that the lockdown coefficient is statistically significant and positive with a magnitude of 0.084 (95% CI: 0.05–0.12; *P* < 0.001). This means that the likelihood of respondents being unable to access medical treatment was 8.4% higher during the lockdown relative to the nonlockdown periods. Because 6.9% of households could not access medical treatment in the nonlockdown period (rounds 3–6), this implies a 122% increase in the share of respondents unable to access healthcare during the lockdown.

**Table 2:** Effect of lockdown on healthcare inaccessibility and unaffordability

Independent variables	(1)	(2)	(3)	(4)
	Unable to access medical treatment	Unable to access medical treatment	Cannot afford medical treatment	Cannot afford medical treatment
Lockdown	0.084***	–	0.044***	–
(95% CI)	(0.049 to 0.118)	–	(0.011 to 0.076)	–
*P* value	0.000	–	0.008	–
L1	–	0.123***	–	0.058***
(95% CI)	–	(0.082 to 0.163)	–	(0.021 to 0.094)
*P* value	–	0.000	–	0.002
L2	–	0.040**	–	0.028
(95% CI)	–	(0.002 to 0.077)	–	(−0.008 to 0.063)
*P* value	–	0.037	–	0.123
COVID-19 cases/100,000	0.003*	0.003	0.003	0.003
(95% CI)	(−0.001 to 0.008)	(−0.001 to 0.007)	(−0.001 to 0.007)	(−0.001 to 0.007)
*P* value	0.096	0.165	0.121	0.149
Number of household members	−0.015	−0.013	−0.013	−0.013
(95% CI)	(−0.035 to 0.005)	(−0.033 to 0.008)	(−0.031 to 0.004)	(−0.030 to 0.005)
*P* value	0.150	0.220	0.130	0.155
Number of observations	7,452	7,452	7,452	7,452
Number of households	2,041	2,041	2,041	2,041

L1 = initial lockdown; L2 = later lockdown. Linear model with household fixed effects. Standard errors are in parentheses and are clustered at the household level. All estimations control for COVID-19 cases and the number of household members.

*** Significance at the 1% level.

** Significance at the 5% level.

* Significance at the 10% level.

Column 1 provides the average effect of the two periods corresponding to the initial, stringent phase and the later, less severe phase of the lockdown. In column 2 of Table 2, we separate the lockdown into the two phases for a more nuanced understanding of their differing effects. We find a significant and positive impact of both lockdown periods on healthcare inaccessibility. Individuals had a 12.3% (95% CI: 0.08–0.16; *P* < 0.001) higher likelihood of being unable to access medical treatment during the stringent phase of lockdowns than nonlockdown periods. During the less strict lockdown the effect was smaller, with an increase of 4.0% (95% CI: 0.0–0.08; *P* < 0.05) over the nonlockdown survey rounds.

There was a statistically significant increase of 4.4% (95% CI: 0.01–0.08; *P* < 0.01) in the likelihood of respondents reporting not being able to access medical treatment during the lockdown because of the lack of money relative to the nonlockdown periods, as shown in column 3 of Table 2. This implies a 71% increase in the share of respondents unable to access healthcare because of unaffordability compared with the nonlockdown period, considering that the average percentage of respondents who reported unaffordability is 6.2% in the nonlockdown period. When we separate the lockdown into the initial and later periods, we find that unaffordability was a problem during the initial stricter phase of the lockdown but not in the later relaxed phase. There was a statistically significant increase of 5.8% (95% CI: 0.02–0.09; *P* < 0.01) in the likelihood of respondents not receiving medical treatment because of financial issues during the initial period, but the effect in the second period was not significantly different from zero (column 4, Table 2).

In addition to the questions about medical treatment, the UHFS also asked the respondents whether any household member could purchase medicines in the past week. However, this was not followed by questions on why the respondent could not buy medicines. Because healthcare access includes access to medicines, we examined the impact of the lockdown on accessibility to medicines. We find an increase in the likelihood of respondents being unable to access medicines after the lockdown. The results are presented in Supplemental Table 1. These results must be interpreted cautiously because the reference period of 1 week for the medicine question places those interviewed in the latter half of round 2 outside the lockdown period.

### Heterogeneity analysis.

Next, we examine whether the effect of the lockdown on the inaccessibility and unaffordability of medical treatment varies by household assets, which proxies for wealth status. Wealthier households should be better positioned to mitigate the financial shock experienced during the lockdown, making it less likely that a lack of money for direct payments will prevent them from accessing healthcare.

Because the UHFS did not have information on assets, we incorporated the asset data from the 2019–2020 UNPS from which the phone survey sample was constructed. In the 2019–2020 UNPS, households reported the value of a wide range of assets owned. A limitation of using prior wealth data is that it could have changed by the time the pandemic started. We use the number of household members living in that survey round to calculate the per-capita asset value for each household. In Table 3, we interact per-capita asset valuation with the lockdown variables and find that households with more assets were not differentially affected compared with those with fewer assets. We also conducted similar heterogeneity analyses on the basis of whether households were rural or urban, whether they relied on agricultural activities for their primary income, and whether they were headed by a male or female (results are presented in Supplemental Tables 2–4). We found no statistically significant differences in the results based on these three characteristics of the households either.

**Table 3:** Differential effect of lockdown on healthcare inaccessibility and unaffordability by asset ownership

	(1)	(2)	(3)	(4)
Independent variables	Unable to access medical treatment	Unable to access medical treatment	Cannot afford medical treatment	Cannot afford medical treatment
Lockdown	0.089***	–	0.047***	–
(95% CI)	(0.053 to 0.125)	–	(0.014 to 0.080)	–
*P* value	0.000	–	0.005	–
L1	–	0.129***	–	0.060***
(95% CI)	–	(0.088 to 0.170)	–	(0.023 to 0.097)
*P* value	–	0.000	–	0.001
L2	–	0.043**	–	0.033*
(95% CI)	–	(0.003 to 0.082)	–	(−0.004 to 0.069)
*P* value	–	0.034	–	0.080
Lockdown × per-capita assets	−0.311	–	0.353	–
(95% CI)	(−1.168 to 0.547)	–	(−0.441 to 1.146)	–
*P* value	0.478	–	0.383	–
L1 × per-capita assets	–	−0.378	–	0.465
(95% CI)	–	(−1.621 to 0.866)	–	(−0.798 to 1.728)
*P* value	–	0.551	–	0.470
L2 × per-capita assets	–	−0.084	–	0.312
(95% CI)	–	(−1.255 to 1.086)	–	(–0.702 to 1.327)
*P* value	–	0.888	–	0.546
COVID-19 cases/100,000	0.005**	0.004*	0.004**	0.004**
(95% CI)	(0.000 to 0.009)	(−0.000 to 0.008)	(0.000 to 0.008)	(0.000 to 0.008)
*P* value	0.030	0.062	0.035	0.045
Number of household members	−0.012	−0.010	−0.011	−0.011
(95% CI)	(−0.032 to 0.007)	(−0.030 to 0.010)	(−0.029 to 0.006)	(−0.028 to 0.007)
*P* value	0.220	0.319	0.194	0.227
Number of observations	7,125	7,125	7,125	7,125
Number of households	1,937	1,937	1,937	1,937

L1 = initial lockdown; L2 = later lockdown. Linear model with household fixed effects. Standard errors are in parentheses and are clustered at the household level. All estimations control for COVID-19 cases and the number of household members.

*** Significance at the 1% level.

** Significance at the 5% level.

* Significance at the 10% level.

### Sensitivity analysis.

Because our estimation relies on matching responses provided during the first and second survey rounds with the lockdown periods, one possible concern is that respondents contacted toward the end of the first and second rounds, which each lasted anywhere from 2 to 3 weeks, could have been describing their healthcare behavior outside the lockdown period. For example, consider a respondent during the second round who was interviewed on August 18, 2020, and replied yes to whether anyone in the household needed medical care on or after June 20, 2020. They could be referring to an illness that occurred on June 30, 2020, during the lockdown, or on August 7, 2020, outside the lockdown period.

To alleviate this concern, we conducted a heterogeneity analysis by grouping respondents by weeks of survey administration. The results are reported in Table 4. The main effects of the lockdown on the inaccessibility of medical treatment continue to hold across all but the last week of the second round. Because a small portion (8.6%) of the second round of interviews took place in the final week of the survey, the statistical insignificance and the opposite sign on the estimated coefficients may be an issue of statistical power.

**Table 4:** Effect of lockdown on healthcare inaccessibility and unaffordability by interview weeks

	(1)	(2)
Independent variables	Unable to access medical treatment	Cannot afford medical treatment
Lockdown round 1: interview days 1–6	0.121***	0.044*
(95% CI)	(0.060 to 0.181)	(−0.005 to 0.092)
*P* value	0.000	0.077
Lockdown round 1: interview days 7–12	0.133***	0.078***
(95% CI)	(0.081 to 0.185)	(0.027 to 0.130)
*P* value	0.000	0.003
Lockdown round 1: interview days 13–19	0.088**	0.021
(95% CI)	(0.003 to 0.172)	(−0.052 to 0.094)
*P* value	0.043	0.579
Lockdown round 2: interview days 1–7	0.045*	0.020
(95% CI)	(−0.001 to 0.090)	(−0.022 to 0.062)
*P* value	0.053	0.343
Lockdown round 2: interview days 8–14	0.060**	0.054**
(95% CI)	(0.014 to 0.106)	(0.008 to 0.100)
*P* value	0.011	0.021
Lockdown round 2: interview days 15–21	−0.072	−0.039
(95% CI)	(−0.204 to 0.060)	(−0.158 to 0.081)
*P* value	0.287	0.525
COVID-19 cases/100,000	0.003	0.003
(95% CI)	(−0.001 to 0.007)	(−0.001 to 0.007)
*P* value	0.158	0.151
Number of household members	−0.013	−0.013
(95% CI)	(−0.033 to 0.008)	(−0.030 to 0.005)
*P* value	0.224	0.159
Number of observations	7,452	7,452
Number of households	2,041	2,041

Linear model with household fixed effects. Standard errors are in parentheses and are clustered at the household level. All estimations control for COVID-19 cases and the number of household members.

*** Significance at the 1% level.

** Significance at the 5% level.

* Significance at the 10% level.

Some of the results on affordability are statistically insignificant depending on the timing of the interview. The impact of the lockdown on unaffordability is statistically significant and positive for interviews conducted from days 1 to 12 of round 1. That is, those interviewed soon after the strictest part of the lockdown were more likely to face unaffordability issues than those interviewed later. Because only 11.9% of round 1 interviews occurred in the last week (days 13–19), this again may be an issue of statistical power.

## DISCUSSION

In this analysis, we find that the lockdown in Uganda from March to August 2020 increased the inaccessibility of medical treatment. The prior literature suggests several factors that could affect the accessibility of treatment during lockdowns, such as travel-related challenges, affordability issues, and supply-side constraints. From a policy perspective, it is important to understand the impact of the above-stated reasons to gauge the efficacy of lockdowns as a public health tool. The goal of this study is to assess the role of unaffordability as a reason for not accessing healthcare.

We find that the dominant reason underlying this inability to access healthcare was the lack of money. Whereas the lockdown was associated with a 122% increase in the share of households that were unable to access medical treatment in times of need relative to nonlockdown periods, it was associated with a 71% increase in the share of households that were unable to afford the treatment. As the restrictions on social contact were gradually relaxed in Uganda, accessibility continued to be a problem, although to a lesser extent. The problem of unaffordability decreased during the lockdown as restrictions were eased.

Our findings on the negative impact of the lockdown in Uganda on access to healthcare services align with the existing evidence from an interrupted time-series study from the country.^11^ However, to our knowledge, we provide the first evidence of the critical role of the lack of money during the lockdown in disrupting healthcare utilization. The lack of money can disrupt healthcare utilization along several dimensions, such as out-of-pocket payment for care services, the cost of medicines and diagnostics, and transportation expenses. Unfortunately, the survey information was not detailed enough to quantify the relative importance of the individual channels.

A study using the same panel data from Uganda found that the COVID-19 lockdown led to substantial declines in paid work, including employment and self-used agricultural work, and income.^46^ Other studies also found significant declines in household income with the pandemic and the lockdown in Uganda.^29^^,^^47^ This in turn would make it difficult for households to afford medical treatment when needed. Households responded to the reduced incomes by decreasing their food intake, increasing their labor supply, selling productive assets, drawing on their savings, and borrowing more. We find that another way of coping with the income shock for some households was to reduce the utilization of healthcare services.

We also find that households with more asset holdings, which we use as a proxy for wealth, were not less likely to report being unable to receive and unable to afford medical treatment after the lockdown. The same is true for households in urban areas—where there is prior evidence of higher catastrophic health expenditures^24^—and households reliant on agriculture. This suggests that people’s access to medical treatment was negatively affected during the lockdown in Uganda, regardless of their wealth, type of employment, and location. The lockdown is, therefore, likely to have maintained the preexisting inequity in access to services in Uganda.^48^^,^^49^ This result is troubling, mainly because the poor and vulnerable already experience a more significant disease burden in low- and middle-income countries (LMICs) as a result of their socioeconomic circumstances.^50^^,^^51^

In the face of potential long-lasting declines in healthcare use and the resulting reversal of decades of progress in improving health outcomes in LMICs because of COVID-19 and the associated lockdowns,^52^^,^^53^ it is crucial for policymakers to consider strategies to reduce the adverse impact of future health crises. Although there have been suggestions of investing in healthcare systems and strengthening effective public health control and surveillance measures,^54^ our results highlight the need for immediate social support for households. Although Uganda announced a nationwide cash transfer plan in July 2021,^55^ our estimates suggest a quicker response would have been more helpful.

Cash transfer programs in LMICs generally improve the utilization of healthcare services in the short and medium runs.^56^ By June 2020, 191 countries had adopted cash transfer programs to counter the COVID-19 crisis,^57^ but future rigorous examination of their transfer modality, targeting mechanism, conditionalities, and impact on healthcare utilization can offer further guidance to governments.

There are two main potential limitations of this study. First, there might be limitations of our fixed-effects model. Although we control for potential time-varying factors that may bias our estimations, the implementation of government programs or the occurrence of other events correlated to variables of interest could have biased our results. Although measurement error is unlikely in the UHFS given the short recall periods, any errors in the outcome variables that are not nested within households^58^ can also be problematic. Furthermore, if the lockdown itself resulted in household members becoming sick and needing care, then the issue of reverse causality could bias our results.^58^

Second, we lack information on the type of medical services households could not access when they needed treatment. Although the survey question about the need for healthcare does not distinguish between COVID and non-COVID services, we believe that most reports of illnesses of household members were non-COVID related because, at the end of the study period, the cumulative rate of COVID-19 cases in Uganda was only 0.09%.^59^ Furthermore, the availability of information on types of non-COVID healthcare services would have enabled us to provide a more nuanced understanding of the lockdown on healthcare utilization. Even more importantly, our results likely underestimate the adverse long-term effects on health because the access questions were only asked if treatment was needed. Given the significant reduction in the use of healthcare when treatment was required, it is likely that preventive care was affected even more. Specifically, households were likely to forego elective, but essential, healthcare uses, such as childhood vaccinations. At best, this means that children, especially those in poor families, will suffer worse future health outcomes. At worst, it allows for the significant spread of infectious diseases that we have made considerable progress against, such as measles.^60^ Despite the limitations of the paper, we report a reduction in healthcare access after the lockdown in Uganda and that the lack of money may be driving it.

## Supplemental Materials


Supplemental materials
