# Supplementary material for: Impacts of the COVID-19 Lockdown on Healthcare Inaccessibility and Unaffordability in Uganda
Source: Am J Trop Med Hyg. 2023 Aug 14;109(3):527–35. doi: 10.4269/ajtmh.23-0144 (PMC10484254; doi:10.4269/ajtmh.23-0144)
Supplement: Supplementary file 1 [file tpmd230144.SD1.pdf]

Supplementary Materials

Table S1: Effect of lockdown on ability to buy medicine in the last week

	**Unable to buy medicine**	
	**(1)**	**(2)**
Lockdown (L)	0.178***	
(95% CI)	(0.143 - 0.212)	
(p value)	0.000	
Lockdown: initial (L1)		0.182***
(95% CI)		(0.142 - 0.222)
(p value)		0.000
Lockdown: later (L2)		0.172***
(95% CI)		(0.129 - 0.215)
(p value)		0.000
Covid-19 cases/100,000	0.007***	0.007***
(95% CI)	(0.004 - 0.011)	(0.003 - 0.011)
(p value)	0.000	0.000
Number of household members	-0.005	-0.004
(95% CI)	(-0.025 - 0.015)	(-0.024 - 0.016)
(p value)	0.646	0.670
No of observations	9,687	9,687
Number of households	2,186	2,186

Notes: Linear model with household fixed effects. Standard errors are in parentheses and are clustered at the household level. All estimations control for Covid-19 cases and number of household members. ***Significance at the 1% level. **Significance at the 5% level. *Significance at the 10% level.

Table S2: Differential effect of lockdown on healthcare inaccessibility & unaffordability by urban-rural

	(1)	(2)	(3)	(4)
	**Unable to access medical treatment**	**Unable to access medical treatment**	**Cannot afford medical treatment**	**Cannot afford medical treatment**
Lockdown (L)	0.077***		0.034*	
(95% CI)	(0.039 - 0.115)		(-0.002 - 0.071)	
(p value)	0.000		0.065	
Lockdown: initial (L1)		0.113***		0.041**
(95% CI)		(0.068 - 0.158)		(0.001 - 0.081)
(p value)		0.000		0.044
Lockdown: later (L2)		0.036*		0.026
(95% CI)		(-0.005 - 0.077)		(-0.015 - 0.066)
(p value)	\	0.086		0.219
L x Urban	0.027		0.038	
(95% CI)	(-0.030 - 0.084)		(-0.012 - 0.088)	
(p value)	0.354		0.135	
L1 x Urban		0.036		0.064*
(95% CI)		(-0.043 - 0.116)		(-0.007 - 0.136)
(p value)		0.373		0.078
L2 x Urban		0.015		0.010
(95% CI)		(-0.047 - 0.077)		(-0.040 - 0.060)
(p value)		0.643		0.692
Urban-dummy variable	0.130	0.120	0.089	0.083
(95% CI)	(-0.136 - 0.396)	(-0.134 - 0.375)	(-0.198 - 0.376)	(-0.195 - 0.361)
(p value)	0.337	0.354	0.544	0.558
Covid-19 cases/100,000	0.003*	0.003	0.003	0.003
(95% CI)	(-0.001 - 0.008)	(-0.001 - 0.007)	(-0.001 - 0.007)	(-0.001 - 0.007)
(p value)	0.096	0.165	0.121	0.150
Number of household members	-0.014	-0.012	-0.013	-0.012
(95% CI)	(-0.035 - 0.006)	(-0.033 - 0.008)	(-0.030 - 0.005)	(-0.030 - 0.006)
(p value)	0.167	0.239	0.157	0.184
No of observations	7,452	7,452	7,452	7,452

Number of households	2,041	2,041	2,041	2,041

Notes: Linear model with household fixed effects. Standard errors are in parentheses and are clustered at the household level. All estimations control for Covid-19 cases and number of household members. ***Significance at the 1% level. **Significance at the 5% level. *Significance at the 10% level

Table S3: Differential effect of lockdown on healthcare inaccessibility & unaffordability by employment type

	**(1)**	**(2)**	**(3)**	**(4)**
	**Unable to access medical treatment**	**Unable to access medical treatment**	**Cannot afford medical treatment**	**Cannot afford medical treatment**
Lockdown (L)	0.080***		0.053**	
(95% CI)	(0.037 - 0.124)		(0.012 - 0.094)	
(p value)	0.000		0.012	
Lockdown: initial (L1)		0.122***		0.072***
(95% CI)		(0.070 - 0.174)		(0.024 - 0.120)
(p value)		0.000		0.004
Lockdown: later (L2)		0.033		0.032
(95% CI)		(-0.016 - 0.082)		(-0.012 - 0.075)
(p value)	\	0.181		0.154
L x Ag household	0.006		-0.020	
(95% CI)	(-0.054 - 0.066)		(-0.073 - 0.034)	
(p value)	0.846		0.476	
L1 x Ag household		-0.000		-0.031
(95% CI)		(-0.076 - 0.076)		(-0.097 - 0.035)
(p value)		0.999		0.354
L2 x Ag household		0.013		-0.008
(95% CI)		(-0.054 - 0.080)		(-0.069 - 0.053)
(p value)		0.702		0.794
Ag household dummy	-0.009	-0.005	0.003	0.004
(95% CI)	(-0.053 - 0.034)	(-0.049 - 0.038)	(-0.039 - 0.045)	(-0.038 - 0.046)
(p value)	0.676	0.805	0.903	0.848
Covid-19 cases/100,000	0.004*	0.003	0.003	0.003
(95% CI)	(-0.000 - 0.008)	(-0.001 - 0.007)	(-0.001 - 0.007)	(-0.001 - 0.007)
(p value)	0.083	0.150	0.122	0.151

Number of household members	-0.015	-0.013	-0.014	-0.013
(95% CI)	(-0.035 - 0.005)	(-0.033 - 0.008)	(-0.031 - 0.004)	(-0.030 - 0.005)
(p value)	0.150	0.219	0.130	0.152
No of observations	7,452	7,452	7,452	7,452
Number of households	2,041	2,041	2,041	2,041

Notes: Linear model with household fixed effects. Standard errors are in parentheses and are clustered at the household level. All estimations control for Covid-19 cases and number of household members. ***Significance at the 1% level. **Significance at the 5% level. *Significance at the 10% level

Table S4: Differential effect of lockdown on healthcare inaccessibility & unaffordability by female headed households

	**(1)**	**(2)**	**(3)**	**(4)**
	**Unable to access medical treatment**	**Unable to access medical treatment**	**Cannot afford medical treatment**	**Cannot afford medical treatment**
Lockdown (L)	0.092***		0.059***	
(95% CI)	(0.055 - 0.128)		(0.023 - 0.094)	
(p value)	0.000		0.001	
Lockdown: initial (L1)		0.127***		0.070***
(95% CI)		(0.082 - 0.171)		(0.030 - 0.110)
(p value)		0.000		0.001
Lockdown: later (L2)		0.051**		0.045**
(95% CI)		(0.010 - 0.091)		(0.004 - 0.086)
(p value)	\	0.014		0.030
Lockdown (L) x Female	-0.026		-0.042	
(95% CI)	(-0.087 - 0.034)		(-0.097 - 0.013)	
(p value)	0.399		0.139	
Lockdown: initial (L1) x Female		-0.014		-0.032
(95% CI)		(-0.093 - 0.065)		(-0.103 - 0.038)
(p value)		0.728		0.370
Lockdown: later (L2) x Female		-0.034		-0.049*
(95% CI)		(-0.100 - 0.031)		(-0.107 - 0.008)
(p value)		0.307		0.093

Female-dummy variable	-0.050	-0.036	0.126	0.130
(95% CI)	(-0.240 - 0.140)	(-0.218 - 0.147)	(-0.060 - 0.312)	(-0.051 - 0.312)
(p value)	0.606	0.700	0.185	0.159
Covid-19 cases/100,000	0.003*	0.003	0.003	0.003
(95% CI)	(-0.001 - 0.008)	(-0.001 - 0.007)	(-0.001 - 0.007)	(-0.001 - 0.007)
(p value)	0.099	0.167	0.114	0.140
Number of household members	-0.015	-0.013	-0.013	-0.012
(95% CI)	(-0.035 - 0.005)	(-0.033 - 0.007)	(-0.030 - 0.004)	(-0.029 - 0.005)
(p value)	0.145	0.216	0.134	0.162
No of observations	7,452	7,452	7,452	7,452
Number of households	2,041	2,041	2,041	2,041

Notes: Linear model with household fixed effects. Standard errors are in parentheses and are clustered at the household level. All estimations control for Covid-19 cases and number of household members. ***Significance at the 1% level. **Significance at the 5% level. *Significance at the 10% level
